# Supplementary material for: A nascent peptide code for translational control of mRNA stability in human cells
Source: Nat Commun. 2022 Nov 11;13:6829. doi: 10.1038/s41467-022-34664-0 (PMC9652226; doi:10.1038/s41467-022-34664-0)
Supplement: Supplementary file 1 — Supplementary Information [file 41467_2022_34664_MOESM1_ESM.pdf]

Supplementary Information

A Nascent Peptide Code for Translational Control of mRNA Stability in Human Cells

Phillip C. Burke[1,2], Heungwon Park[1], Arvind Rasi Subramaniam[1*]

[1] Basic Sciences Division and Computational Biology Section of the Public Health Sciences Division, Fred Hutchinson Cancer Center, Seattle, WA 98109, USA [2] Department of Microbiology, University of Washington, Seattle, WA 98195, USA

[*] Corresponding author: rasi@fredhutch.org

Supplementary Figure 1

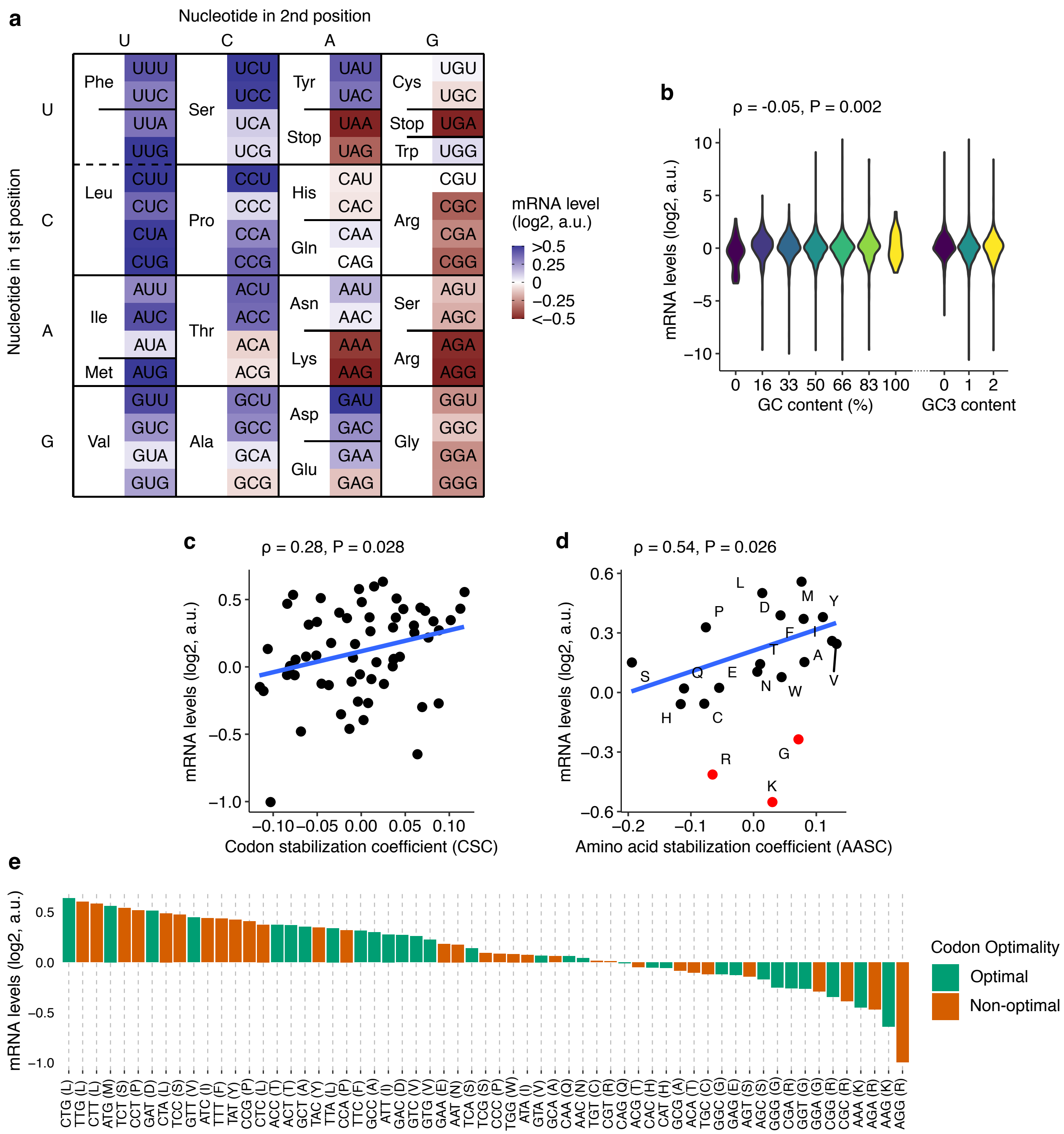

(a) Heatmap of mean effect of each codon on mRNA levels averaged across both positions of 8x dicodon repeat. Values >0.50 were set to 0.51, and values <-0.5 were set to -0.51 to highlight differences in intermediate values. **(b)** mRNA levels of dicodon repeats as a function of their GC content (left) or the number of GC3s in the dicodon (right). Spearman rank correlation coefficient ρ and its P-value are shown for GC content (two-sided t-test); GC3 content had no significant correlation with mRNA levels. **(c)** Average codon effects on mRNA levels as a function of their mean codon stabilization coefficient[1]. Pearson correlation coefficient ρ and its P-value are shown (two-sided t-test). **(d)** Average amino acid effects on mRNA levels of as a function of their mean amino acid stabilization coefficient[2]. Pearson correlation coefficient ρ and it's P-value are shown, calculated from the points in black. Points in red (arginine, lysine, and glycine) are excluded from this Pearson correlation calculation. **(e)** Average codon effects on mRNA levels plotted from high to low, with codons colored by HEK293T codon optimality[1].

Supplementary Figure 2

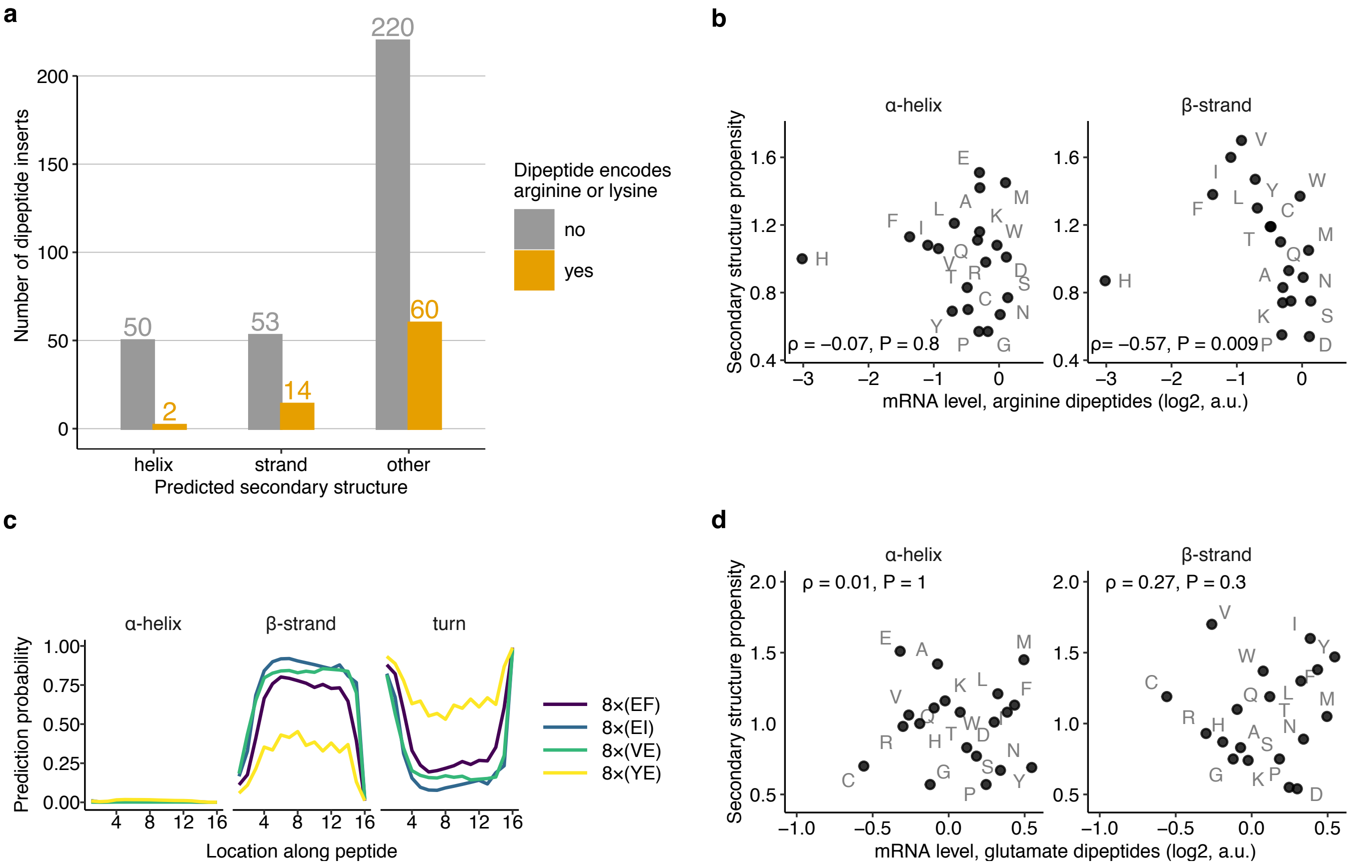

(a) Number of dipeptide-encoding reporters from Fig. 1c that contain either arginine or lysine amino acids, partitioned by predicted protein secondary structure as in Fig. 3b.
(b,d) mRNA levels of dipeptide repeat-encoding reporters with arginine (b) or glutamate (d) in one position and one of twenty amino acids in the other position of the repeat (labeled in grey) plotted as a function of the propensity[3] of the second amino acid to occur in a β strand (right) or an α helix (left). ρ is the Spearman correlation coefficient between the two axes with the indicated *P* value.
(c) Computationally predicted secondary structure probability along 16 amino acid-long peptide sequences encoded by dipeptides with glutamate that form β strands. Secondary structure probabilities are predicted using S4PRED[4].
Amino acids are labeled by their one-letter codes.

Supplementary Figure 3

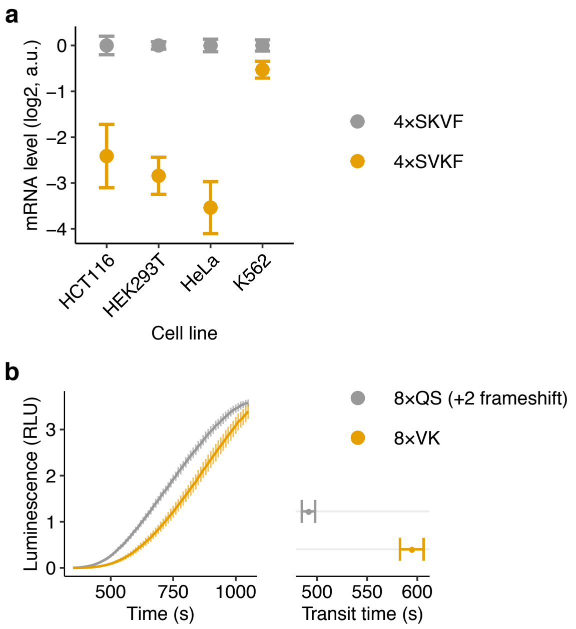

(a) mRNA levels of reporters with α helix $(SKVF)_4$ or β strand $(SVKF)_4$ forming peptides in 4 different cell lines HCT116, HEK293T, HeLa, and K562. Data are presented as mean values and error bars represent standard error of measurement over a median of 550 barcodes per insert calculated using 100 bootstrap samples. **(b)** *In vitro* measurement of ribosome transit time on mRNAs encoding the 8xVal-Lys dipeptide or its +2 frameshifted control, followed by Nanoluciferase. Luminescence is measured as a function of time after addition of *in vitro* transcribed mRNAs to rabbit reticulocyte lysate (RRL) at t=0*s* (left panel). Standard error of measurement across three technical replicates is shown as a shaded area on either side of the mean. Ribosome transit times (right panel) are estimated by measuring the X-intercept of the linear portion of the raw luminescence signal in the left panel.

Supplementary Figure 4

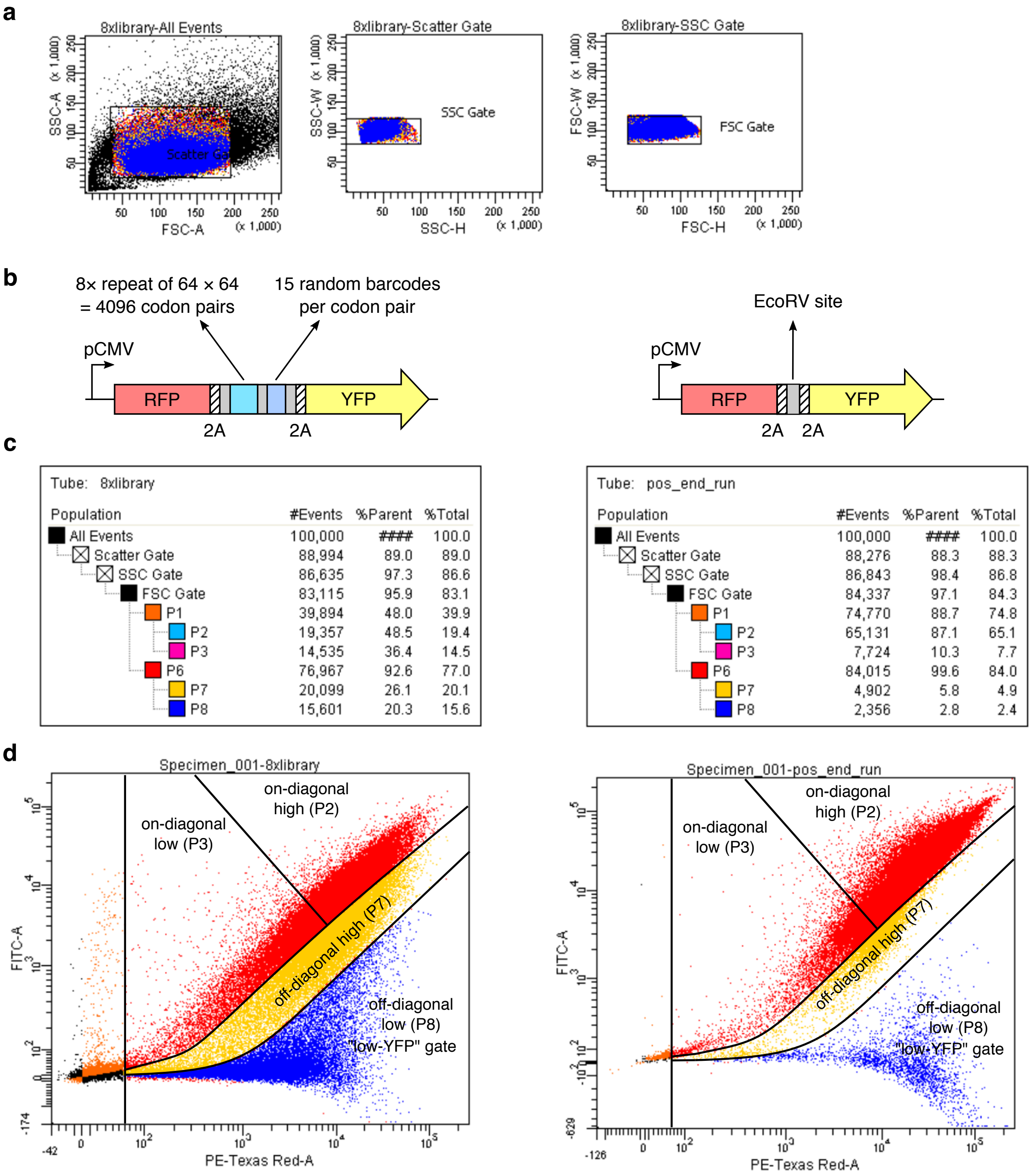

Tube: 8xlibrary

Population	#Events	%Parent	%Total
All Events	100,000	####	100.0
Scatter Gate	88,994	89.0	89.0
SSC Gate	86,635	97.3	86.6
FSC Gate	83,115	95.9	83.1
P1	39,894	48.0	39.9
P2	19,357	48.5	19.4
P3	14,535	36.4	14.5
P6	76,967	92.6	77.0
P7	20,099	26.1	20.1
P8	15,601	20.3	15.6

Tube: pos_end_run

Population	#Events	%Parent	%Total
All Events	100,000	####	100.0
Scatter Gate	88,276	88.3	88.3
SSC Gate	86,843	98.4	86.8
FSC Gate	84,337	97.1	84.3
P1	74,770	88.7	74.8
P2	65,131	87.1	65.1
P3	7,724	10.3	7.7
P6	84,015	99.6	84.0
P7	4,902	5.8	4.9
P8	2,356	2.8	2.4

(a) Nested FACS-seq gating strategy for the integrated 8× dicodon library from Fig.1. Forward and side scatter gates (SSC-A vs. FSC-A, SSC-W vs. SSC-H, and FSC-W vs. FSC-H).
(b) Schematics of the 8× dicodon plasmid library (pPBHS286, "8xlibrary", left panel in **c**) and the parent vector control reporter (pPBHS285, "pos_end_run", right panel in **c**).
(c) Percentage of cells binned into each FACS-seq gate, for 100,000 measured cells, for the 8× dicodon library and parent vector cell lines.
(d) FACS-seq RFP (PE.Texas.Red.A) and YFP (FITC.A) gate settings for the library and parent vector cell lines. Gates were set manually during sorting based on RFP and YFP expression levels and RFP/YFP ratios, aiming to sort a roughly equal percentage of total 8× dicodon library cells into each of the four populations denoted "On-diagonal High", "On-diagonal Low", "Off-Diagonal High", and "Off-diagonal Low". The "Off-diagonal Low" gate corresponds to the "low-eYFP" gate indicated in Fig. 2c. Approximately 2.5 million cells were sorted per gate. Barcode sequencing counts are available for all 8× dicodon library gates, as well as unsorted 8× dicodon library cells from the same cell suspension used for FACS.

Supplementary References:

1. Wu, Q. *et al.* Translation affects mRNA stability in a codon-dependent manner in human cells. *eLife* **8**, e45396 (2019).

2. Forrest, M. E. *et al.* Codon and amino acid content are associated with mRNA stability in mammalian cells. *PLoS ONE* **15**, e0228730 (2020).

3. Chou, P. Y. & Fasman, G. D. Empirical Predictions of Protein Conformation. *Annu. Rev. Biochem.* **47**, 251–276 (1978).

4. Moffat, L. & Jones, D. T. Increasing the accuracy of single sequence prediction methods using a deep semi-supervised learning framework. *Bioinformatics* **37**, 3744–3751 (2021).
