# Supplementary material for: A nascent peptide code for translational control of mRNA stability in human cells
Source: Nat Commun. 2022 Nov 11;13:6829. doi: 10.1038/s41467-022-34664-0 (PMC9652226; doi:10.1038/s41467-022-34664-0)
Supplement: Supplementary file 3 — Description of Additional Supplementary Files [file 41467_2022_34664_MOESM3_ESM.pdf]

Description of Additional Supplementary Files

File Name: Supplementary Data 1
Description: List of Oligos, Plasmids, and Cell Lines.
