# Supplementary material for: A nascent peptide code for translational control of mRNA stability in human cells
Source: Nat Commun. 2022 Nov 11;13:6829. doi: 10.1038/s41467-022-34664-0 (PMC9652226; doi:10.1038/s41467-022-34664-0)
Supplement: Supplementary file 5 — Reporting Summary [file 41467_2022_34664_MOESM5_ESM.pdf]

nature portfolio

Corresponding author(s): Arvind Rasi Subramaniam

Last updated by author(s): Oct 26, 2022

Reporting Summary

Nature Portfolio wishes to improve the reproducibility of the work that we publish. This form provides structure for consistency and transparency in reporting. For further information on Nature Portfolio policies, see our Editorial Policies and the Editorial Policy Checklist.

Statistics

For all statistical analyses, confirm that the following items are present in the figure legend, table legend, main text, or Methods section.

n/a	Confirmed	
☐	☒	The exact sample size (*n*) for each experimental group/condition, given as a discrete number and unit of measurement
☐	☒	A statement on whether measurements were taken from distinct samples or whether the same sample was measured repeatedly
☐	☒	The statistical test(s) used AND whether they are one- or two-sided<br>*Only common tests should be described solely by name; describe more complex techniques in the Methods section.*
☒	☐	A description of all covariates tested
☒	☐	A description of any assumptions or corrections, such as tests of normality and adjustment for multiple comparisons
☐	☒	A full description of the statistical parameters including central tendency (e.g. means) or other basic estimates (e.g. regression coefficient) AND variation (e.g. standard deviation) or associated estimates of uncertainty (e.g. confidence intervals)
☐	☒	For null hypothesis testing, the test statistic (e.g. *F*, *t*, *r*) with confidence intervals, effect sizes, degrees of freedom and *P* value noted<br>*Give P values as exact values whenever suitable.*
☒	☐	For Bayesian analysis, information on the choice of priors and Markov chain Monte Carlo settings
☒	☐	For hierarchical and complex designs, identification of the appropriate level for tests and full reporting of outcomes
☐	☒	Estimates of effect sizes (e.g. Cohen's *d*, Pearson's *r*), indicating how they were calculated

Our web collection on statistics for biologists contains articles on many of the points above.

Software and code

Policy information about availability of computer code

Data collection	High-throughput sequencing data were collected using Illumina MiSeq, HiSeq 2500, and NextSeq 2000 platforms. FACS data was collected using BD FACSDiva Software.
Data analysis	Data were analyzed using Bowtie2 (v2.4.2), Python (v3.6.2), and R (v3.7.4). The R packages tidyverse (v1.3.2), biostrings (v2.62.0), and plotrix (v3.8-2) were used. Code is available on Github at https://github.com/rasilab/burke_2022

For manuscripts utilizing custom algorithms or software that are central to the research but not yet described in published literature, software must be made available to editors and reviewers. We strongly encourage code deposition in a community repository (e.g. GitHub). See the Nature Portfolio guidelines for submitting code & software for further information.

Data

Policy information about availability of data

All manuscripts must include a data availability statement. This statement should provide the following information, where applicable:

- Accession codes, unique identifiers, or web links for publicly available datasets
- A description of any restrictions on data availability
- For clinical datasets or third party data, please ensure that the statement adheres to our policy

All high throughput sequencing data is publicly available at NCBI as part of BioProject PRJNA785998. All other data are available in a public GitHub repository (https://github.com/rasilab/burke_2022).

Human research participants

Policy information about studies involving human research participants and Sex and Gender in Research.

Reporting on sex and gender	n/a
Population characteristics	n/a
Recruitment	n/a
Ethics oversight	n/a

Note that full information on the approval of the study protocol must also be provided in the manuscript.

Field-specific reporting

Please select the one below that is the best fit for your research. If you are not sure, read the appropriate sections before making your selection.

☒ Life sciences ☐ Behavioural & social sciences ☐ Ecological, evolutionary & environmental sciences

For a reference copy of the document with all sections, see nature.com/documents/nr-reporting-summary-flat.pdf

Life sciences study design

All studies must disclose on these points even when the disclosure is negative.

Sample size	Library sizes were chosen such that each variable insert tested was covered by at least 10 barcodes and these redundant libraries were maintained at >100x coverage throughout the cloning and selection steps. In each case sample size was appropriate based on the consistency of measurable differences between groups.
Data exclusions	No data were excluded from analyses.
Replication	For barcode sequencing, error bars were calculated as the standard deviation of 100 bootstrap samples of barcodes across the gRNA and mRNA samples. Barcode sequencing measurements for plasmid library pools 1, 3, and 4 were performed as single experiments. Plasmid library pool 2 was generated and tested in independent biological duplicate. Circular dichroism measurements were performed independently twice at the 0% TFE, and once at 20% and 40% TFE. Luciferase assays were performed in technical triplicate, on two biologically independent inserts per secondary structure condition tested. All attempts at replication were successful and are reported in the manuscript.
Randomization	Samples were not assigned to different cohorts or study arms, so randomization would not apply to the work done in this study. All samples were treated the same.
Blinding	No group allocation or blinding was performed in this study.

Reporting for specific materials, systems and methods

We require information from authors about some types of materials, experimental systems and methods used in many studies. Here, indicate whether each material, system or method listed is relevant to your study. If you are not sure if a list item applies to your research, read the appropriate section before selecting a response.

Materials & experimental systems

n/a	Involved in the study	
☒	☐	Antibodies
☐	☒	Eukaryotic cell lines
☒	☐	Palaeontology and archaeology
☒	☐	Animals and other organisms
☒	☐	Clinical data
☒	☐	Dual use research of concern

Methods

n/a	Involved in the study	
☒	☐	ChIP-seq
☐	☒	Flow cytometry
☒	☐	MRI-based neuroimaging

Eukaryotic cell lines

Policy information about cell lines and Sex and Gender in Research

Cell line source(s)	All line lines in this study were obtained from ATCC. HEK293T cells (RRID:CVCL_0063, ATCC CRL-3216), HCT116 cells (RRID:CVCL_0291, NCI60 cancer line panel), Hela cells (RRID:CVCL_0030, ATCC CCL-2), K562 cells (RRID:CVCL_0004, ATCC CCL-243).
Authentication	HEK293T cells and Hela cell identities were confirmed by short tandem repeat analysis. Other cell lines were not authenticated. Only low passage cell lines (directly from ATCC) were used, to avoid cross-contamination or phenotypic change upon culture.
Mycoplasma contamination	All cell lines were confirmed negative for mycoplasma contamination.
Commonly misidentified lines (See ICLAC register)	No misidentified cell lines were used in the study.

Flow Cytometry

Plots

Confirm that:

☒ The axis labels state the marker and fluorochrome used (e.g. CD4-FITC).

☒ The axis scales are clearly visible. Include numbers along axes only for bottom left plot of group (a 'group' is an analysis of identical markers).

☒ All plots are contour plots with outliers or pseudocolor plots.

☒ A numerical value for number of cells or percentage (with statistics) is provided.

Methodology

Sample preparation	Two 15 cm dishes of 75% confluent hsPB80 cells stably expressing the pHPHS286 library were used as input for fluorescence-activated cell sorting.
Instrument	BD FACSAria II flow cytometer
Software	Data was collected and analyzed using BD FACSDiva Software. Cell population data was also plotted using R (analysis code available on GitHub)
Cell population abundance	2.5M cells with ~10-fold or greater RFP expression relative to YFP were sorted into the low-YFP gate. Number and percentage of cells sorted into each gate, shown for 100,000 events, in are provided in Fig. S5.
Gating strategy	Fluorescence gates were determined using hsPB80 cells containing the pHPHS285 no-insert parent vector and untransfected hsPB80 cells as positive and negative controls for RFP and YFP fluorescence. Full gating strategy for the pHPHS286 library cells and pHPHS285 no-insert cells is in Fig S5.

☒ Tick this box to confirm that a figure exemplifying the gating strategy is provided in the Supplementary Information.
